# Recurrent multicentric peripheral ossifying fibroma-like lesion in a child: a case report

**DOI:** 10.1186/1756-0500-7-673

**Published:** 2014-09-25

**Authors:** Marina Deus Moura Lima, Rubens G Teixeira, Marcelo Bonecker, Paulo de Camargo Moraes, Andrea Mantesso

**Affiliations:** University Federal of Piauí – UFPI, Campus Universitário Ministro Petrônio Portella, Bloco 5, Teresina-PI, 64049-550 Brazil; São Leopoldo Mandic Dental Research Institute, Rua José Rocha Junqueira 13, Perdizes, Campinas-SP 13045-755 Brazil; School of Dentistry, University of São Paulo, Av Professor Lineu Prestes, 2227, Cidade Universitária, São, Paulo-SP 05508-000 Brazil

**Keywords:** Gingival Hyperplasia, Peripheral ossifying fibroma, Multicentric, Children

## Abstract

**Background:**

Gingival hyperplasias are peculiar conditions that may produce extreme growth that impairs masticatory function and causes psychological and aesthetic disturbances. They can vary from mild interdental papillae localized growth to marked swelling affecting both jaws.

**Case presentation:**

The aim of this case report is to present a rare case of generalized gingival growth diagnosed in a 4 year-old Caucasian child and followed for 9 years. The lesion covered almost all of the upper and lower teeth and recurred thirty times with the same clinical and histopathological aspects. The clinical features suggested the diagnosis of idiopathic gingival fibromatosis, but the histopathological aspects did not confirm this hypothesis and were consistent with peripheral ossifying fibroma.

**Conclusion:**

The present case reports a rare gingival growth with challenging diagnosis and treatment.

## Background

Gingival enlargements are a heterogeneous group of disorders characterized by progressive enlargement of the gingiva and caused by an increase in submucosal connective tissue elements. Its etiology and pathogenesis are not fully understood, but it can associate with local factors such as dental plaque and caries, individual susceptibility and the action of substances and their metabolites. They can be iatrogenic, inherited or idiopathic. The condition is not painful until the tissue enlarges to partially cover the occlusal surface and becomes traumatized during mastication [1, 2].

Peripheral ossifying fibroma (POF) is a benign, well-demarcated lesion composed of fibrocellular tissue and mineralized material of varying appearance such as bone, cementum, or both [3]. It occurs predominantly in the second decade of life, typically measures less than 1.5 cm in diameter, is commonly ulcerated and/or pink to red in color, and normally appears as a solitary and slow-growing nodular mass that can be either pedunculated or sessile [3–6]. The etiology of POF remains undetermined, but the most accepted idea is that it is a reactive process. The lesion is probably initiated by local irritants occurring in the gingival crevice or associated with adjacent teeth [7, 8].

Our case shows an extensive multicentric recurrent lesion with an aberrant amount of bone and cementum-like calcifications. We discuss our findings and compare the behavior and clinical aspects of our case to typical cases of idiopathic gingival fibromatosis (IGF) and Peripheral ossifying fibroma (POF).

## Case presentation

A 4-year-old Caucasian boy was referred to our clinic with a complaint of an extensive lesion inside his mouth.

**Figure 1:**
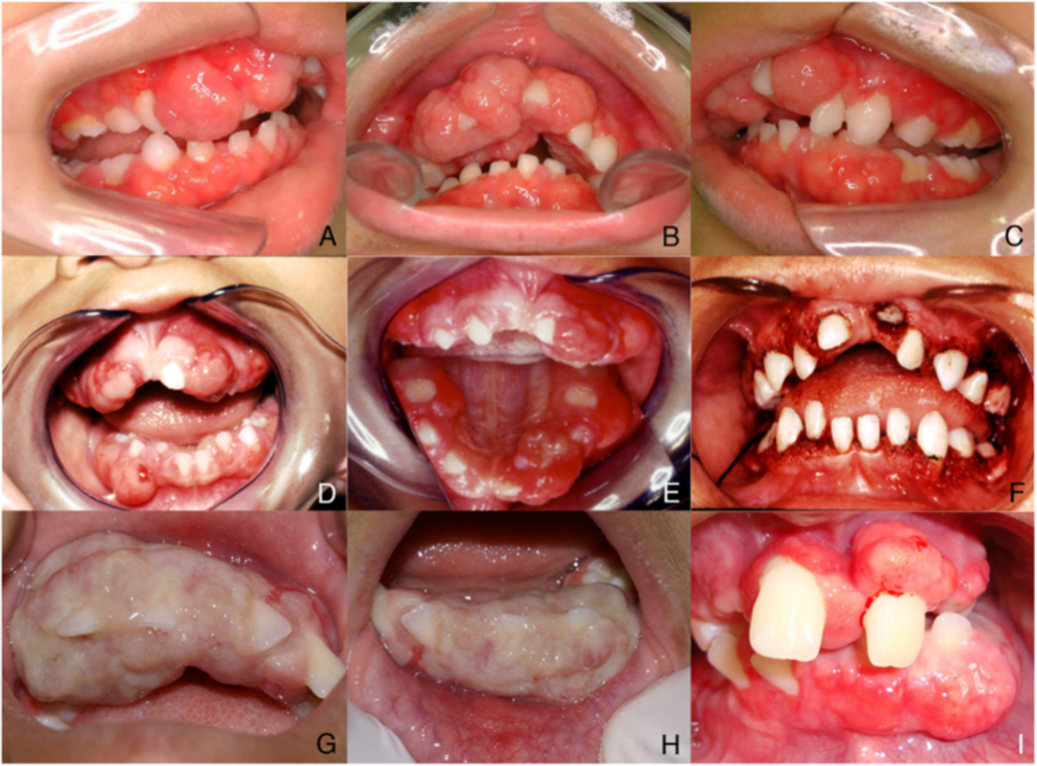
**Clinical images.** Intra-oral examination showing an extensive lesion covering the teeth of the maxilla and mandible **(A, B, C)**. Intra-oral view of the second recurrence observed 8 months after the first surgery **(D)**. One year after the second recurrence, a new growth could be observed **(E)** and was surgically removed once more **(F)**; Clinical aspect of another recurrence when the patient was 11 years old. Note the lingual and vestibular surfaces showing an extensive gingival growth. The gingiva at that moment appeared white in color **(G, H)**. A recent intraoral view exhibiting one more recurrence **(I)**.

Extra-oral examination revealed that his cheeks appeared to be slightly swollen. The patient was unable to close his mouth due to interior swelling.Intra-oral examination revealed an extensive exophytic, sessile, firm lesion with areas of ulcerated surface and a pink-to-red coloration that covered almost all the upper and lower teeth (Figures 1A, B, C). The lesion was painless but was interfering with masticatory function. The mother mentioned that the patient was 8 weeks premature, was 2.2 kg at birth, had not been breast-fed, and was always at the lower limit of the growth curve. At 4 years old, the patient weighed 18 kg and was 107 cm tall. The patient’s mother related that she had to take growth hormones for many years until she reached adulthood. According to her, no other member of the patient’s family had any kind of gingival growth.

Laboratory tests performed before the initial biopsy included hemogram, immunoglobulins, growth hormone (GH), parathormone and glycosaminoglycan levels, and tissue plasminogen activity. The results were normal and the patient was not under any kind of medication.

**Figure 2:**
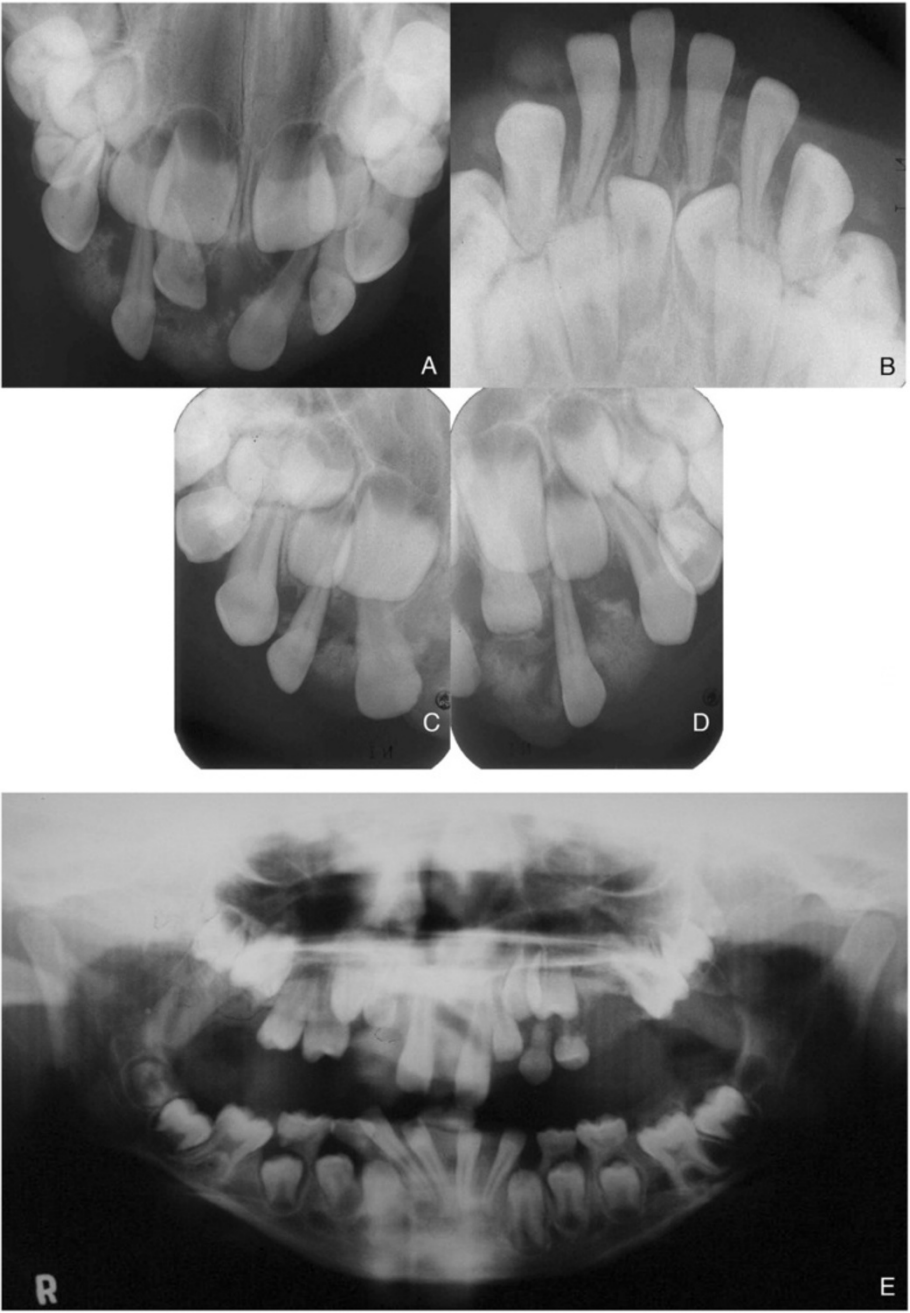
**Radiographs of the case.** Upper and lower occlusal radiographs and periapical radiographs presenting an explosive pattern of peripheral mineralization affecting different areas of the maxilla and mandible **(A, B, C, D)**. Panoramic radiograph taken when patient was 8 years old, revealing the presence of another peripheral mineralized growth and the dentition development with an irregular chronology. Note the agenesis of the second upper bicuspid tooth **(E)**.

**Figure 3:**
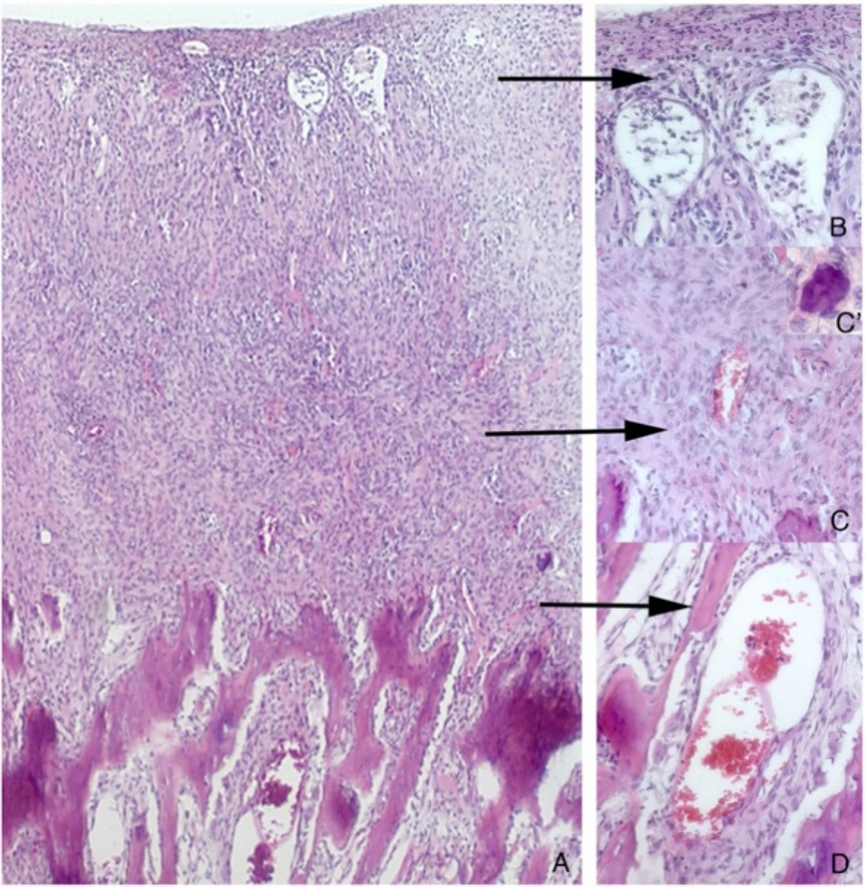
**Histological sections stained with H.E.** Histopathological aspect of POF exhibiting the high cellular area on the top and the extensive mineralization on the bottom of the field. Note that the mineralized area showed bone trabeculae organized parallel to each other, and there was a clear distinction between the mineralized and more cellular areas **(A)**. The top of the gingival growth was frequently ulcerated with presence of inflammatory cells (arrow) **(B)**; the proliferating cells were big, round (arrow) and no clear distinction could be seen among them, **(C)**. Nodules of cementum could be often observed **(C’)**. Bone trabeculae were immature, presenting osteoblasts in their periphery (arrow). This area was rich in blood vessels **(D)**.

Establishing a clinical differential diagnosis was a complex process. Osteosarcoma, peripheral ossifying fibroma, idiopathic gingival fibromatosis, hereditary gingival fibromatosis, plasminogen deficiency, mucopolysaccharidosis II, ligneous conjunctivitis, exostosis, and Gardner syndrome were checked for.Occlusal, panoramic, and periapical radiographs showed external radiopaque extensive lesion in a pattern of explosive mineralization restricted to the gingiva, without affecting the bone (Figures 2A-E).An incisional biopsy was performed, and the histological analysis revealed a local atrophic stratified squamous epithelium or ulceration area overlying fibrovascular connective tissue with strips of calcified material (Figures 3A, B, C, C’,D). The lesion had a high cellularity and a formation of bone located mostly at the periphery (Figure 3A). Occasionally, cementum-like calcifications were observed (Figure 3C’). No sign of cellular atypia could be found. With the histopatological diagnosis of a peripheral ossifying fibroma-like lesion and under general anesthesia, the remaining mass was removed, including the periosteum. In addition, the patient was given educational instructions, motivation, fluoride applications, and professional removal of dental plaque.Eight months later, the patient returned and a complete recurrence of the lesion was observed (Figure 1D). He underwent another surgery and the specimen was sent for a histopathological study with a diagnosis of recurrent peripheral ossifying fibroma-like lesion. One year after the second surgery, he had a third recurrence (Figure 1E) and was operated on again (Figure 1F). The patient was forwarded for evaluation with a geneticist to verify the possibility of any syndrome associated with the gingival growth, but no genetic alteration was found.Two years later, he had a fourth recurrence, and, 5 months after the removal of this lesion, he had a fifth recurrence. When he was 11 years old, he had a complete recurrence that covered all of his upper and lower teeth (Figures 1G, H). Presently, the patient is 13 years-old and the multicentric gingival growth is again present in his mouth (Figure 1I). During a period of 9 years, the lesion recurred about 30 times, showing the same clinical, radiographic, and histopathological aspects (Figure 1D-I).

The histological pattern of the lesions removed were the same in all recurrences. Usually full mouth gingivectomies were performed by conventional approach using Kirkland and orban periodontal knives, electro-section or electro-coagulation methods under local anesthesia. The surgeries were performed in quadrants taking care to remove the involved periodontal ligament and periosteum. As an adjuvant for treatment, corticosteroid infiltration or trichloroacetic acid (TCA) peeling were used in association with some removals, but no alteration in the development of new recurrences was noted.

## Discussion

The differential diagnosis of gingival growths include a variety of lesions such as fibro-osseous lesions like peripheral ossifying fibromas and juvenile ossifying fibromas and connective tissue lesions such as pyogenic granulomas and idiopathic gingival fibromatosis. Due to the age of our patient and the aggressive appearance, aggressive lesions such as osteosarcoma and central juvenile ossifying fibroma were first considered. However, the X-Ray images clearly demonstrated that the lesion was restricted to the gingiva and did not affect the bone besides the rare superficial erosion forming a cupping defect in focal areas. In our case, because of the long evolution and the clinical and radiographic aspects, both osteosarcoma and juvenile ossifying fibroma were excluded. Pyogenic granuloma was never considered because of the abundant presence of mineralization.

Some other alterations were also surveyed. The initial suspect was idiopathic gingival fibromatosis, but the color and consistency of the gum and the histopathological characteristics were not consistent with this diagnosis. A possibility of hereditary gingival fibromatosis was discarded early on in anamneses because there was no family history of this disease. Besides, histopathologically, both idiopathic gingival fibromatosis and hereditary gingival fibromatosis have different characteristics from peripheral ossifying fibroma.

We also suspected plasminogen deficiency, but the patient’s levels of plasminogen were normal. The possibility of mucopolysaccharidosis II (Hunter syndrome) was discussed. However, the patient did not have any of the systemic signs of the accumulation of GAGs, which generally include macrocephaly, dental abnormalities, hyperactivity, hepatosplenomegaly, mental retardation, or chronic diarrhea, among others. Ligneous conjunctivitis can cause periodontal lesions similar to those exhibited by our patient, but it is a serious systemic disease that has other manifestations, such as recurrent chronic conjunctivitis, that were clearly not present. The possibility of exostosis and Gardner syndrome was discarded, as it was clear that the lesions were of gingival origin. Other manifestations of Gardner syndrome such as osteomas in sinuses and epidermoid cysts on the skin were also not present.

The type of mineralized component presented in POF is variable and can consist of bone, cementum-like calcification, or distrophic calcification. Frequently, there is a combination of these mineralized materials [4]. In the present case, in rare areas, bone and cementum-like calcifications were observed, but the position and the pattern of the bone was noted to be quite unusual. Mineralization was always close to the alveolar bone, with the mineralized trabeculae in a parallel pattern starting from the base of the lesion towards its surface. This pattern of explosive mineralization is very common in aggressive lesions such as osteosarcomas, but in the present case, no sign of a malignancy such as cellular atypia or high number of mitosis have been found in any of the recurrences over the years. In addition, this explosive growth pattern was limited to a small area seen in the first biopsy while the majority of the lesion and the recurrences showed a classic peripheral ossifying fibroma histopathological appearance. Therefore, due to the typical histopathological aspect associated with the uncommon multicentric characteristic and frequent recurrences, a diagnosis of peripheral ossifying fibroma-like lesion was established.

Histologically, idiopathic gingival fibromatosis is mainly due to an increase and thickening of mature collagen bundles in the connective tissue stroma. The nodular appearance can be attributed to the thickened para hyperkeratinized epithelium. It can be seen in conjunction with growth hormone deficiency and may exhibit presence of dystrophic calcification. None of our patient’s lesions showed the histopathological aspect of a gingival fibromatosis, as they were mainly formed by robust round cells and showed areas of mineralization as bone and cementum nodules. It did not show collagen accumulation.

The clinical characteristics of the lesion directed us to a possible idiopathic gingival fibromatosis, however, histological characteristics that were repeated in the multiple recurrences pushed us away from that possibility. A variety of histological samples were sent to 3 independent pathologists during the 9 years of the recurrences, and all 3 were definite in the histological diagnosis of POF. Therefore, we chose to nominate this lesion as a peripheral ossifying fibroma-like lesion based on its histopathological aspect.

Nonetheless, it is not possible to discriminate whether ours is a case of idiopathic gingival fibromatosis atypical for the overproduction of bone and cementum or a case of peripheral ossifying fibroma atypical by its numerous recurrences and multicentric nature.

To our knowledge, only one case of a multicentric peripheral ossifying fibroma has been published in the literature to date [7]. This case consisted of recurrent lesions, measuring from 5 mm to 3 cm in diameter, observed in the maxillary and mandibular gingiva of a 49-year-old female who complained of pain and bleeding after palpation. She had her lesions removed by simple excision and electrosurgery over the years with subsequent recurrences. Antibiotic and intra-lesional steroid therapies were also used with no response. Like our patient, this other case of multicentric peripheral ossifying fibroma had many laboratory tests performed with unremarkable results.

The similarities shared between these two cases are the constant recurrences and affection of multiple areas. However, our multicentric recurrent case appears to be more aggressive, since the lesions commonly covered almost all of the teeth from the maxilla and mandible.

The patient’s mother had to be treated with the growth hormone during her infancy. GH is essential for bone formation and remodeling, not only in childhood but also in adulthood. *In vitro* studies have shown that GH stimulates osteoblast function and proliferation both directly and indirectly by local stimulation of GF induction [9]. Although it is not possible to establish a correlation between this fact and the patient’s condition, a genetic predisposition to bone alterations may be possible. It is important to point out that the levels of GH in our patient were within normal limits. The multiple and recurrent pattern of the condition and the absence of any alteration in the laboratorial and radiographic exams point to a systemic predisposition.

The bacterial plaque must be considered as an etiological factor capable of predisposing to the appearance of the peripheral ossifying fibroma lesions [4]. Therefore, the patient has been under professional care in regards to regular teeth cleaning since the first recurrence, and he was taught how to proceed with buccal hygiene in a correct and careful way. After the beginning of the professional preventive procedures, such as dental plaque removal, the patient still had recurrences, and the lesions were similar to the previous ones, affecting both the maxilla and mandible. Close professional supervision made the prompt identification of the lesions easier, but even in the beginning of their development the recurrences were always multicentric, affecting both the maxilla and mandible.

It has been previously discussed that the surgical technique employed is associated with the probability of recurrences of peripheral ossifying fibroma, and a total excision of the involved periodontal ligament and periosteum is required to minimize it [4]. Moreover, regardless of the surgical technique employed, it is important to eliminate etiological factors including plaque, calculus, and plaque-retention restorations [4]. In our case, despite the careful surgical technique used in all surgeries with the removal of the involved periosteum, the lesion recurred around thirty times.

## Conclusion

The present case report is an atypical multicentric recurrent peripheral ossifying fibroma-like lesion that offers challenges in the diagnosis and treatment due to its frequent recurrences.

### Consent

Written informed consent was obtained from the patient’s parents for publication of this Case Report and any accompanying images. A copy of the written consent is available for review by the Editor-in-Chief of this journal.

## Abbreviations

POF: Peripheral ossifying fibroma
BSP: Bone sialoprotein
TCA: Trichloroacetic acid
GH: Growth hormone.
